# Omega-3 Fatty Acid-Type Docosahexaenoic Acid Protects against Aβ-Mediated Mitochondrial Deficits and Pathomechanisms in Alzheimer’s Disease-Related Animal Model

**DOI:** 10.3390/ijms21113879

**Published:** 2020-05-29

**Authors:** Yong Ho Park, Soo Jung Shin, Hyeon soo Kim, Sang Bum Hong, Sujin Kim, Yunkwon Nam, Jwa-Jin Kim, Kyu Lim, Jong-Seok Kim, Jin-il Kim, Seong Gak Jeon, Minho Moon

**Affiliations:** 1 Department of Biochemistry, College of Medicine, Konyang University, 158, Gwanjeodong-ro, Seo-gu, Daejeon 35365, Korea; znf900809@naver.com (Y.H.P.); tlstnzz83@gmail.com (S.J.S.); sooya1105@naver.com (H.s.K.); harryhong0314@gmail.com (S.B.H.); aktnfl3371@naver.com (S.K.); yunkwonnam@gmail.com (Y.N.); 2 Department of Nephrology, School of Medicine, Chungnam National University, Daejeon 35015, Korea; kjj1021@naver.com; 3 Department of Biochemistry, School of Medicine, Chungnam National University, Daejeon 35015, Korea; kyulim@cnu.ac.kr; 4 Myunggok Medical Research Institute, College of Medicine, Konyang University, Daejeon 35365, Korea; jskim7488@konyang.ac.kr; 5 Department of Nursing, College of Nursing, Jeju National University, Jeju-si 63243, Korea; 6 Biopharmaceutical Chemistry Major, School of Applied Chemistry, Kookmin University, Seongbuk-gu, Seoul 02707, Korea

**Keywords:** Alzheimer’s disease, mitochondria, omega-3, DHA, fat-1 mice, 5XFAD mice

## Abstract

It has been reported that damage to the mitochondria affects the progression of Alzheimer’s disease (AD), and that mitochondrial dysfunction is improved by omega-3. However, no animal or cell model studies have confirmed whether omega-3 inhibits AD pathology related to mitochondria deficits. In this study, we aimed to (1) identify mitigating effects of endogenous omega-3 on mitochondrial deficits and AD pathology induced by amyloid beta (Aβ) in fat-1 mice, a transgenic omega-3 polyunsaturated fatty acids (PUFAs)-producing animal; (2) identify if docosahexaenoic acid (DHA) improves mitochondrial deficits induced by Aβ in HT22 cells; and (3) verify improvement effects of DHA administration on mitochondrial deficits and AD pathology in B6SJL-Tg(APPSwFlLon,PSEN1*M146L*L286V)6799Vas/Mmjax (5XFAD), a transgenic Aβ-overexpressing model. We found that omega-3 PUFAs significantly improved Aβ-induced mitochondrial pathology in fat-1 mice. In addition, our in vitro and in vivo findings demonstrate that DHA attenuated AD-associated pathologies, such as mitochondrial impairment, Aβ accumulation, neuroinflammation, neuronal loss, and impairment of adult hippocampal neurogenesis.

## 1. Introduction

Alzheimer’s disease (AD) is one of the most prevalent forms of dementia, responsible for more than half of all dementia cases. The etiology of AD is not yet fully understood, but the build-up of amyloid-beta (Aβ) has been reported to be the most significant pathological hallmark [1]. Aβ aggregates consist of various types, such as dimers and oligomers, among which oligomers are known to be the most neurotoxic form through their binding of various receptors [2]. Many studies suggest that Aβ aggregates have a toxic effect on neurons by inducing microglia- and astrocyte-associated neuroinflammation [3,4]. In addition to the toxicity of Aβ itself on neurons, interestingly, Aβ induces changes in mitochondrial function, resulting in apoptotic cell death [5].

Correct mitochondrial function is essential for life and is responsible for energy metabolism, cellular survival, and apoptosis in neurodegenerative diseases, including AD. Not only do mitochondria perform these functions, but they can also mediate Aβ peptide-induced damage [6]. Aβ can lead to cell death caused by abnormal mitochondrial gene expression [7], and has been shown to impair mitochondrial function in HT22 mouse hippocampal neuronal cells [8]. This has also been shown in PC12 cells, through decreasing mitochondrial membrane potential, and thereby increasing vulnerability to oxygen and glucose deprivation and inducing reactive oxygen species [9]. Aβ-treated neuronal cells exhibit significantly increased mitochondrial fission. Moreover, Aβ reduces the activities of cytochrome c oxidase, the α-ketoglutarate dehydrogenase complex, and pyruvate dehydrogenase in isolated rat brain mitochondria [10]. Mitochondrial damage results in reduced neuronal ATP production and increased oxidative stress, which is known to cause neurotransmission damage, synaptic damage, loss of synapses, and eventually decline of cognitive functions in patients with AD [7]. Notably, mitochondrial function is associated with the regulation of adult hippocampal neurogenesis, while mitochondrial dysfunction induces cognitive impairment via depletion of neural stem cells [11]. Moreover, several studies have indicated that the mitochondrial metabolism stage-specifically mediates the regulation of adult hippocampal neurogenesis [12].

Contemporary perspectives on AD highlight the importance of mitochondrial cascades. There is strong evidence suggesting a critical role of mitochondrial integrity in AD [13]. Blocking cyclophilin D, a component of mitochondrial permeability transition pore, enhances cognitive functions as well as diminishes oxidative stress in amyloid precursor protein (APP) transgenic mice. This indicates the significance of the interaction between Aβ and the mitochondrial permeability transition pore in AD pathogenesis [14]. In line with these findings, studies using AD models of cytoplasmic hybrid cell lines, such as SH-SY5Y AD cybrids and NT2 AD cybrids, demonstrate alterations in mitochondrial function and morphology [15]. Therapeutic strategies for AD should therefore encompass the regulation of mitochondrial function.

Omega-3 polyunsaturated fatty acids (PUFAs) are classified into alpha-linolenic acid (ALA, c18:3), docosapentaenoic acid (DPA, c22:5), eicosapentaenoic acid (EPA, c20:5), and docosahexaenoic acid (DHA, c22:6). These fatty acids encompass a significant role in maintaining the function of cell membranes [16]. Levels of omega-3 PUFAs, including DHA, a PUFA byproduct, are decreased in the blood plasma of patients with dementia including AD [17]. These have been shown to regulate the formation of synaptic vesicles in cultured mouse fetal hypothalamic cells [18]. In addition, rats with decreased levels of DHA—induced by an omega-3 PUFAs-deficient diet—exhibit reduced olfactory-based cognitive functions [19]. Numerous studies have described the neuroprotective roles of DHA in the brain [20], and cohort studies have also revealed that consumption of DHA or fish containing DHA is associated with a reduced risk of cognitive impairment in middle-aged or aged populations [21,22]. Importantly, several studies have suggested that DHA attenuates cognitive impairment in animal models of AD, and that its protective action may be mediated by reduction of AD-associated pathologies, such as oxidative stress [23,24]. DHA supplementation has also been shown to ameliorate postsynaptic protein loss and behavioral deficits [25], as well as reduce Aβ accumulation in AD transgenic mice [26]. These studies illustrate the therapeutic potential of DHA for the treatment of AD.

Despite the studies assessing the positive effects of omega-3 PUFAs in AD patients [27], to date, there are very few studies examining the effect of DHA on mitochondrial dysfunction and Aβ-related pathologies in AD mouse models. We therefore conducted this study to elucidate the effect of DHA in the pathogenesis of AD and mitochondrial dysfunction. Here, using transgenic fat-1 mice, which can convert omega-6 PUFAs to omega-3 PUFAs endogenously [28], we show that an increased level of endogenous omega-3 PUFAs ameliorates impaired mitochondrial dynamics and neuronal loss induced by Aβ-stereotaxic injection. In addition, we also report that DHA, a byproduct of omega-3 PUFAs, treatment protects against mitochondrial dysfunction in both the HT22 cell line and the transgenic 5XFAD AD model. Furthermore, DHA attenuates Aβ accumulation, neuroinflammation, neuronal loss, and adult hippocampal neurogenesis impairment in B6SJL-Tg(APPSwFlLon,PSEN1*M146L*L286V)6799Vas/Mmjax (5XFAD) mice.

## 2. Results

### 2.1. Cytotoxicity Evaluation of DHA in the HT22 Cell Line

An MTT assay was performed to evaluate DHA cytotoxicity in the HT22 hippocampal cell line. The results indicated that incubation for 24 h and 48 h with increasing concentrations of DHA (1, 10, 20, 40, and 50 μg/mL) did not lead to significant neurotoxicity (Figure S1). Therefore, subsequent experiments were conducted using 10–50 μg/mL DHA for 24 h.

### 2.2. Endogenous Elevation of Omega-3 PUFAs Ameliorates Aβ-Induced Mitochondrial Dynamics Impairment

**Figure 1:**
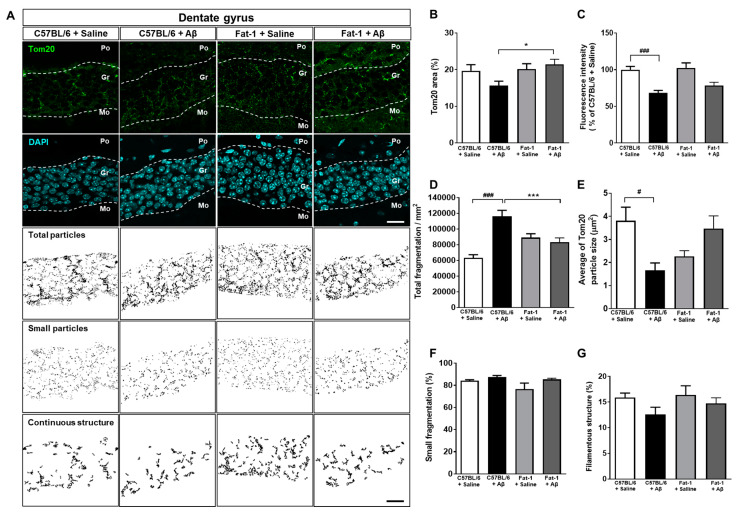
Effects of endogenous elevation of omega-3 PUFAs on altered mitochondrial dynamics demonstrated by Aβ-stereotaxic injection in fat-1 mice. (**A**) Immunofluorescence staining against Tom20 was conducted to examine the mitochondrial dynamics in the dentate gyrus of the hippocampus (scale bar = 20 μm) [8,31]. (**B**) Quantification of the Tom20-positive area plotted as a percentage. (**C**) Quantification of Tom20 fluorescence intensity was normalized to that observed in saline-injected C57BL/6 mice. Total fragmentation (**D**), average Tom20 particle size (**E**), small fragmentation (**F**), and filamentous structures (**G**) were quantified by classifying the immuno-positive area of Tom20 according to the mitochondrial structure sizes, to demonstrate the fusion and fission of mitochondria. Data are presented as mean ± SEM (30–36 images/5–6 mice). ^#^ *p* < 0.05 and ^###^ *p* < 0.001: C57BL/6 + Saline versus C57BL/6 + Aβ. * *p* < 0.05 and *** *p* < 0.001: C57BL/6 + Aβ versus Fat-1 + Aβ. Po: polymorph layer, Gr: granular layer, Mo: molecular layer.

To assess the effects of endogenous elevation of omega-3 PUFAs on Aβ-induced alterations in mitochondrial dynamics, we performed immunohistochemistry to label translocases of mitochondrial outer membrane 20 (Tom20) antibody in the granular layer of the dentate gyrus (DG) of fat-1 mice. The percentage of the Tom20 (+) area and the fluorescence intensity were measured for morphological analysis and quantification of mitochondrial dynamics (Figure 1A). The Tom20 (+) area coverage was significantly larger in Aβ-injected fat-1 mice than in Aβ-injected C57BL/6 mice (Figure 1B). However, the fluorescence intensity of Tom20, which was significantly reduced following stereotaxic injection of Aβ in C57BL/6 mice, was not significantly improved in Aβ-injected fat-1 mice (Figure 1C). Remarkably, the total number of fragments per area, which was significantly increased for Aβ-injected C57BL/6 mice, was significantly reduced in Aβ-injected fat-1 mice (Figure 1D). The average size of the Tom20 particles significantly decreased in the Aβ-injected C57BL/6 mice compared with saline-injected C57BL/6 mice. However, there was increase in the average Tom20 particle size in the Aβ-injected fat-1 mice compared with saline-injected fat-1 mice (Figure 1E). However, the small fragmentation and the filamentous structure were not significantly different between the three groups (Figure 1F,G). Based on previous data indicating higher levels of omega-3 PUFAs in the hippocampus and forebrain of fat-1 mice compared with that of wild-type mice [28,29,30], these data suggest that endogenous elevated omega-3 PUFAs in fat-1 mice could mitigate mitochondrial fragmentation induced by hippocampal Aβ injection.

### 2.3. Endogenous Elevation of Omega-3 PUFAs Reduces Aβ-Induced Neuronal Loss

**Figure 2:**
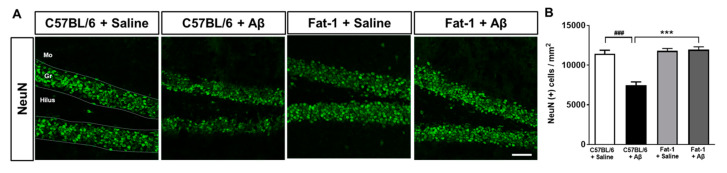
Neuroprotective effects of endogenous elevation of omega-3 PUFAs on neuronal loss demonstrated following Aβ-stereotaxic injection in fat-1 mice. (**A**) Immunofluorescent staining was performed with the neuronal marker (neuronal nuclei) NeuN in the DG (scale bar = 50 μm). (**B**) The number of NeuN-stained cells per area in the granular cell layer of the DG was significantly increased in fat-1 + Aβ mice compared to the number observed in C57BL/6 + Aβ mice. Data are presented as mean ± SEM (30–36 images/5–6 mice). ^###^ *p* < 0.001: C57BL/6 + Saline versus C57BL/6 + Aβ. *** *p* < 0.001: C57BL/6 + Aβ versus Fat-1 + Aβ. Gr: granular layer, Mo: molecular layer.

We examined whether endogenous elevation of omega-3 PUFAs attenuates neuronal death induced by hippocampal injection of Aβ. Immunohistochemical staining was performed for neuronal nuclei (NeuN) in the DG of fat-1 mice (Figure 2A). Neuronal loss induced by Aβ-stereotaxic injection was restored to the C57BL/6 + Saline group levels in the fat-1 + Aβ group (Figure 2B). These results indicate that elevated levels of endogenous omega-3 PUFAs have a protective effect against Aβ-induced neuronal damage.

### 2.4. DHA Attenuates Aβ-Induced Mitochondrial Dysfunction in HT22 Cells

**Figure 3:**
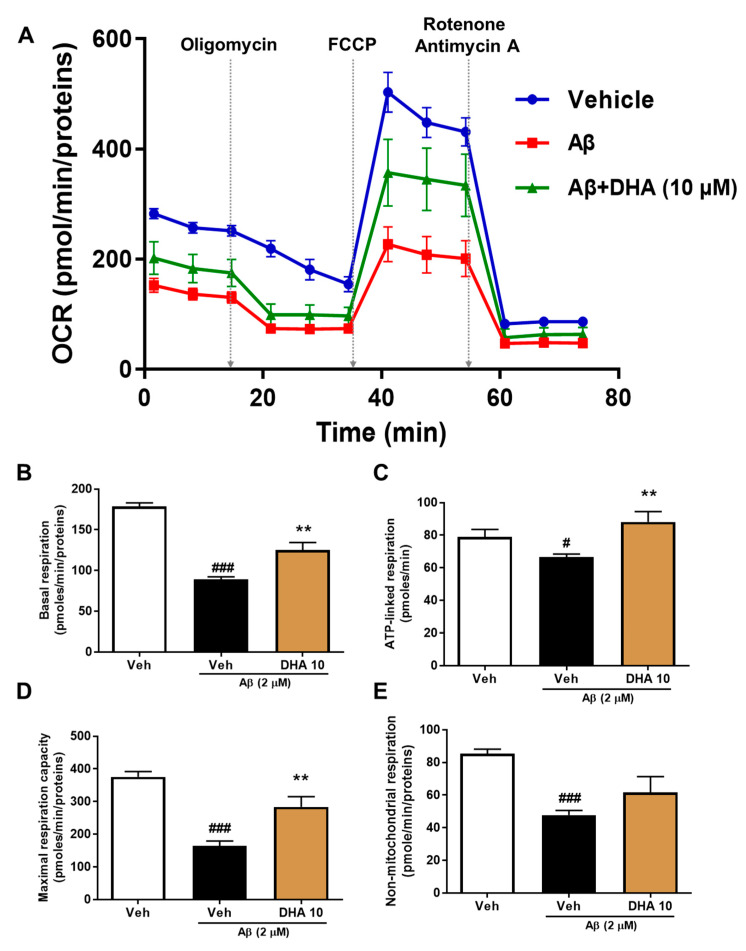
DHA alleviates Aβ-induced mitochondrial dysfunction in HT22 cells. (**A**) The OCR in HT22 hippocampal cell lines was measured using the Seahorse XFp analyzer after treatment with 2 μM Aβ or Aβ + DHA (10 μM). Basal respiration (**B**), ATP-linked reparation (**C**), maximal respiration capacity (**D**), and non-mitochondrial respiration (**E**) were derived by analyzing OCR values following the addition of FCCP (1 μM), oligomycin (1 μM), and rotenone (0.5 μM) + antimycin A (0.5 μM). Data are expressed as mean ± SEM. ^#^ *p* < 0.05 and ^###^ *p* < 0.001: vehicle-treated cells versus Aβ-treated cells. ** *p* < 0.01.

To investigate the effects of DHA, a byproduct of omega-3 PUFAs, on Aβ-induced mitochondrial dysfunction, HT22 cells were incubated without and with DHA (10 μg/mL) in the existence of 2 μM Aβ. The Agilent seahorse XFp analyzer was used to measure the oxygen consumption rate (OCR) (Figure 3A). It was shown that Aβ-treated HT22 cells exhibit a significant decrease in basal respiration, resulting from mitochondrial proton seepage and ATP demand caused by Aβ treatment. DHA treatment recovered this Aβ-induced impairment (Figure 3B). ATP-linked respiration was then measured following a treatment of an adenosine triphosphate synthase inhibitor, oligomycin (1 μM). Aβ treatment also significantly reduced ATP-linked respiration as well as OCR. However, 10 μg/mL DHA restored ATP-linked respiration to control levels (Figure 3C). The physiological energy requirements stimulated by FCCP [carbonyl cyanide 4-(trifluoromethoxy) phenylhydrazone] (2 μM), a mitochondrial uncoupler, determines the maximum respiratory capacity by the maximum OCR level. Treatment with DHA enhanced the maximum respiratory capacity following damage induced by Aβ. We blocked the gradient of hydrogen ions between the mitochondrial matrix and the intermembrane space by adding complex I (rotenone, 0.5 μM) and complex III (antimycin A, 0.5 μM) inhibitors in order to measure persistent OCR levels; this represents non-mitochondrial respiration maintained by a subset of cellular enzymes such as NADPH oxidases, cytochrome P450s, or cyclooxygenases. The non-mitochondrial respiration was also impaired by Aβ and was not rescued by DHA treatment (Figure 3E). Collectively, these results indicate that DHA can attenuate mitochondrial deficits induced by Aβ in an HT22 neuronal cell line.

### 2.5. DHA Protects against the Alteration of Mitochondrial Dynamics in the Dorsal Subiculum of 5XFAD Mice

**Figure 4:**
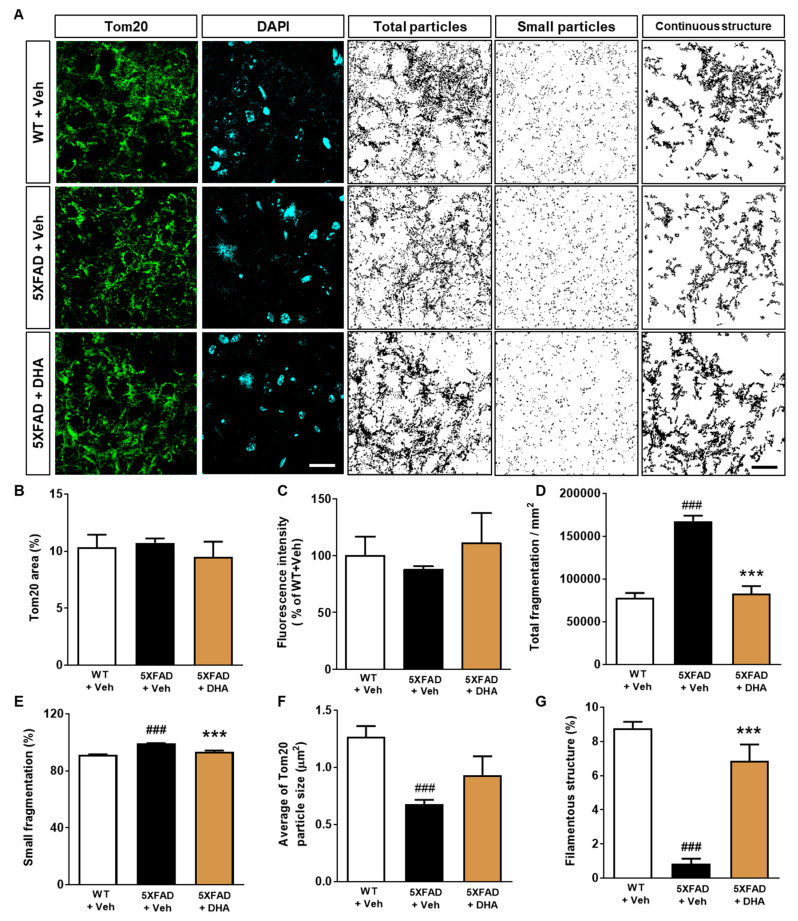
Effects of DHA on mitochondrial dynamics altered by Aβ accumulation in the 5XFAD mice. (**A**) Immunofluorescence staining against Tom20 was performed to visualize the mitochondrial dynamics in the dorsal subiculum (scale bar = 20 μm). (**B**) Fraction of Tom20-positive area plotted as a percentage. (**C**) The fluorescence intensity of Tom20 was normalized to that observed in vehicle-treated WT mice. The immuno-positive area of Tom20 was classified according to the size to demonstrate mitochondrial fusion and fission, to allow quantification of the following factors: total fragmentation (**D**), proportion of small fragmentation (%) (**E**), proportion of filamentous structures (%) (**F**), and average Tom20 particle size (**G**). Data are presented as mean ± SEM (42 images/7 mice). ^###^ *p* < 0.001: vehicle-administrated WT mice versus vehicle-administrated 5XFAD mice. *** *p* < 0.001: vehicle-treated 5XFAD mice vs. DHA-treated 5XFAD mice.

The subiculum is considered to be one of the earliest regions to be affected by Aβ accumulation [32,33] (Figure S2). Accordingly, to investigate whether DHA, a byproduct of omega-3 PUFAs, has an effect on regulating the alteration of mitochondrial dynamics in AD, the dorsal subiculum of 5XFAD mice was immunohistochemically stained with a Tom20 antibody. For quantification of mitochondrial dynamics and morphological analysis, the percentage of the Tom20 (+) areas and the fluorescence intensity of the Tom20 immunoreactivity were measured (Figure 4A). While there were no differences in the fluorescence intensity and Tom20 (+) area between the groups (Figure 4B,C), DHA administration significantly reduced the increased total fragmentation observed in vehicle-treated 5XFAD mice (Figure 4D). The proportion of small fragmentation significantly elevated in the vehicle-treated 5XFAD mice compared with vehicle-treated WT mice. However, proportion of small fragmentation decreased in the DHA-treated 5XFAD mice compared with vehicle-treated 5XFAD mice (Figure 4E). The average Tom20 particle size tended to increase with DHA administration, but the effect was not statistically significant (Figure 4F); however, the proportion of filamentous structures also significantly increased following DHA administration compared to vehicle (Figure 4G). These results suggest that administration of DHA protects the alteration of mitochondrial dynamics in transgenic mice with Aβ overexpression.

### 2.6. DHA Attenuates Aβ Burden in the Dorsal Subiculum of 5XFAD Mice

**Figure 5:**
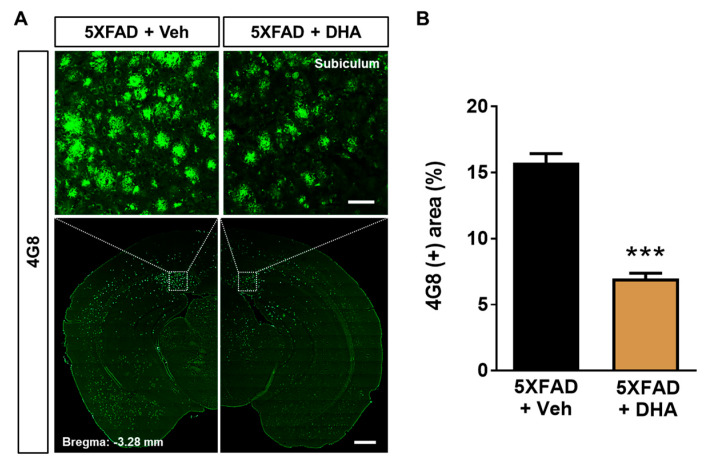
Effects of DHA on Aβ accumulation in the 5XFAD mice. (**A**) Immunofluorescence staining with a 4G8 antibody was performed to visualize the Aβ burden with or without DHA in the dorsal subiculum of 5XFAD mice (upper panel = 50 μm, lower panel = 500 μm). (**B**) 4G8-positive area coverage was significantly attenuated in the dorsal subiculum of DHA-treated 5XFAD mice compared to those in vehicle-treated 5XFAD mice. Data are presented as mean ± SEM (42 images/seven mice). *** *p* < 0.001.

We speculated that mitochondrial protection by DHA treatment (Figure 1, Figure 3 and Figure 4) might also affect Aβ accumulation. In order to demonstrate the effect of DHA on Aβ accumulation, we investigated whether administration of DHA could inhibit Aβ deposition in the 5XFAD mice. Brain sections were stained using a 4G8 antibody to evaluate the Aβ burden (Figure 5A). The 4G8 (+) area coverage was significantly reduced by DHA treatment in the dorsal subiculum of 5XFAD mice (Figure 5B). These results demonstrated that administration of DHA significantly decreases deposition of Aβ in the brain of 5XFAD mice.

### 2.7. DHA Reduces Neuroinflammation and Neuronal Loss in the Dorsal Subiculum of 5XFAD Mice

**Figure 6:**
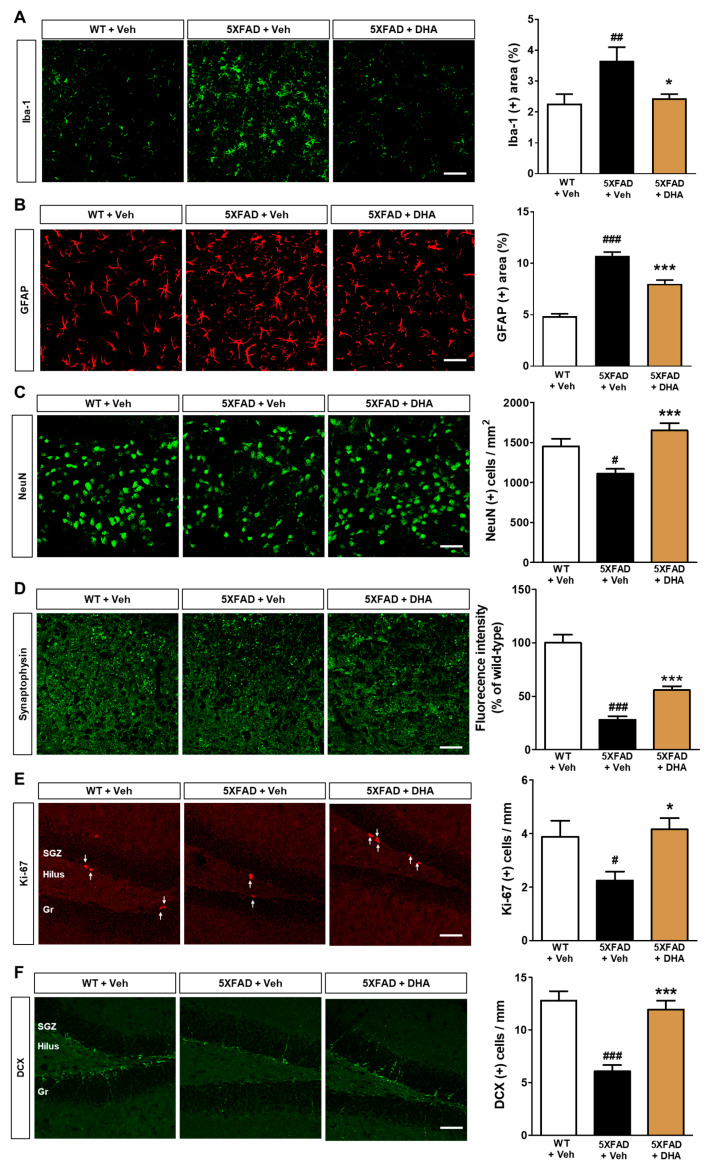
Effects of DHA on AD-related pathogenesis in the 5XFAD mice. (**A**–**D**) Immunofluorescence staining was performed for evaluation of neuroinflammation and neuronal loss with markers of microglia (Iba-1), astrocytes (GFAP), neurons (NeuN), and presynaptic vesicles (SYN) in the dorsal subiculum. (**A**,**B**) The percentage of Iba-1 (+) and GFAP (+) area coverage significantly increased in 5XFAD mice compared to WT mice, but this was significantly alleviated by DHA administration. (**C**) The significant reduction in the number of NeuN (+) cells per area in the dorsal subiculum observed in 5XFAD mice was significantly alleviated by DHA administration. (**D**) The fluorescence intensity of SYN in the dorsal subiculum was normalized to that observed in vehicle-treated WT mice. The reduced SYN fluorescence intensity in 5XFAD mice was significantly relieved by DHA administration. (**E**,**F**) Immunofluorescence staining was performed for the evaluation of adult hippocampal neurogenesis with the proliferating cell marker Ki-67 and the neuronal precursor marker DCX in the SGZ of the DG. A significant reduction in the number of Ki-67 (+) and DCX (+) cells per length of SGZ was observed in 5XFAD mice, and was significantly alleviated by DHA administration. All histological qualitative data are presented as mean ± SEM (scale bar = 50 μm, 42 images/seven mice). ^#^ *p* < 0.05, ^##^ *p* < 0.01, and ^###^ *p* < 0.001: vehicle-administered WT mice versus vehicle-treated 5XFAD mice. ^*^ *p* < 0.05 and ^***^ *p* < 0.001: vehicle-treated 5XFAD mice vs. DHA-treated 5XFAD mice. Gr: granular layer, Mo: molecular layer, SGZ: subgranular zone.

We then investigated whether administration of DHA could protect against neuroinflammation and neuronal cell death observed in 5XFAD mice. Immunohistochemical staining was performed with ionized calcium-binding adapter molecule 1 (Iba-1) and glial fibrillary acidic protein (GFAP) antibodies to label microglia and astrocytes, respectively, in the dorsal subiculum of 5XFAD mice. It was observed that significant increase in Iba-1 (+) and GFAP (+) area coverage in vehicle-treated 5XFAD mice. However, these increases were ameliorated by DHA administration (Figure 6A,B). In addition, the increased numbers of Iba-1 and GFAP-positive cells per area within the hippocampus in 5XFAD mice were also significantly alleviated by DHA administration (Figure S3). We also performed immunohistochemical staining with NeuN and synaptophysin (SYN) antibodies to evaluate neuronal and synaptic loss, respectively. Both the number of NeuN (+) cells and the fluorescence intensity of SYN immunoreactivity were decreased in 5XFAD mice, and were then alleviated by DHA treatment (Figure 6C,D). These results suggest that treatment with DHA can ameliorate neuroinflammation and neurodegeneration in Aβ-overexpressing mice.

### 2.8. DHA Significantly Alleviates Hippocampal Adult Neurogenesis Impairment in 5XFAD Mice

Mitochondria are known to be crucial regulators in adult neurogenesis [12]. Despite the treatment of DHA having been shown to enhance cognitive function in AD animal models [23,24], no evidence has illustrated the effect of DHA on hippocampal neurogenesis. To examine the effect of DHA on the diminished adult hippocampal neurogenesis observed in 5XFAD mice, immunohistochemical staining against Ki-67 and doublecortin (DCX) was conducted for visualization of proliferating cells and neuronal precursors, respectively. The number of Ki-67-positive and DCX-positive cells per length of the subgranular zone (SGZ) significantly decreased in the vehicle-treated 5XFAD mice compared to the vehicle-treated WT mice. However, DHA administration in 5XFAD mice alleviated this and restored proliferation and neural precursor cell numbers to control levels (Figure 6E,F). These results can provide histopathological evidence that demonstrates that DHA enhances adult hippocampal neurogenesis impairment in an AD animal model.

## 3. Discussion

It is known that mitochondrial dysfunction is highly correlated with Aβ accumulation as well as with Aβ-related toxicity [13]. Moreover, there are several studies reporting beneficial effects of omega-3 PUFAs, including effects of the omega-3 fatty acid DHA on Aβ-associated pathologies [23,24]. However, no study to date has studied the effects of DHA on mitochondrial dysfunction in AD. Thus, we investigated the effects of elevated endogenous levels of omega-3 PUFAs and subsequently DHA administration on Aβ-mediated mitochondrial dysfunction and AD pathology. Mitochondrial dysfunction, including aberrant mitochondrial dynamics, is observed in AD and is known to have a critical role in AD pathogenesis. Considering that excessive mitochondrial fission is associated with neurodegenerative conditions, modulating abnormal mitochondrial dynamics could be a potential therapeutic target for AD [34]. In the present study, we observed impaired mitochondrial dynamics in both Aβ-administered C57BL/6 mice and 5XFAD mice. In addition, the endogenous elevation of omega-3 PUFAs and exogenous administration of DHA ameliorated these impaired mitochondrial dynamics. Increased mitochondrial fragmentation in AD brain has been shown to be associated with an elevation in mitochondrial fission and decrease in mitochondrial fusion. Moreover, the interaction between dynamin-related protein 1 and Aβ induces mitochondrial fragmentation and results in neuronal and synaptic damage [35,36]. While no beneficial effects of DHA on mitochondrial dysfunction in AD have been reported so far, one previous study seems to corroborate our findings [37]. This study reported that maternal DHA supplementation increases the hippocampal levels of DHA in the offspring. Furthermore, it also inhibits the increased mitochondrial fission seen in the hippocampus of female offspring following prenatal restraint stress by decreasing oxidative stress [37]. Despite finding no difference in filamentous mitochondrial structures between Aβ-injected C57BL/6 mice and Fat-1 mice, we found that DHA increased the proportion of filamentous structures in the dorsal subiculum of 5XFAD mice. This discrepancy between two different animal models may be due to multiple factors, including the differences in strains, origin of Aβ (exogenous or endogenous), duration of Aβ exposure, or differences between effects of endogenous elevation of omega-3 PUFAs and exogenous administration of DHA. Thus, interpreting the results regarding the effects of DHA should be done with caution. Considering that the damage to mitochondrial structures is a characteristic of Aβ-induced changes in mitochondria [38], our results illustrating structural preservation of mitochondria following DHA administration imply that DHA exerts a protective effect to prevent Aβ-associated mitochondrial dysfunction. Accordingly, fat-1 mice that endogenously produce omega-3 PUFAs that are not usually produced in mammals [28] contain higher levels of DHA than other omega-3 PUFAs, such as EPA and DPA, in the brain [39]. Thus, based on the results of our DHA administration in 5XFAD mice (Figure 4, Figure 5 and Figure 6), it can be speculated that the increased levels of DHA primarily mediate the beneficial effects of elevated endogenous omega-3 PUFAs on mitochondrial dysfunction (Figure 1 and Figure 2).

Aβ-induced mitochondrial dysfunction encompasses not only the alteration of mitochondrial dynamics but also the impairment of mitochondrial respiration [40]. Aβ is known to predominantly inhibit the activity of mitochondrial complex IV in both in vivo and in vitro AD models [41,42]. Moreover, treatment with Aβ significantly reduces the mitochondrial oxygen consumption rate [8]. Notably, the transcellular spread of pathogenic Aβ via extracellular vesicles has been shown to contribute to mitochondrial dysfunction in both AD patients and animal models [43]. Moreover, previous studies have reported that mitochondrial bioenergetic dysfunction occurs even before the onset of AD pathology such as Aβ plaque formation [44]. Aforementioned findings indicate that the enhancement of mitochondrial function could play a protective role in AD pathogenesis. As expected, we observed reduced levels of basal respiration, ATP-linked respiration, maximal respiratory capacity, and non-mitochondrial respiration impairment in Aβ-treated HT22 neuronal cells (Figure 3). In the present study, DHA attenuated the impairment of mitochondrial respiration induced by Aβ in HT22 neuronal cells. Considering that Aβ impairs mitochondrial morphology and membrane potential, and consequently leads to cellular apoptosis [5], the DHA-mediated improvement of mitochondrial respiration highlights its possible therapeutic efficacy in the pathogenesis of AD. Immunohistochemical analysis demonstrated that the administration of DHA attenuates Aβ burden in the dorsal subiculum of 5XFAD mice (Figure 5). The ameliorating effect of DHA on Aβ accumulation has been reported in various models of AD, and many mechanisms have been proposed. Notably, one study showed that DHA is a suppressor of γ-secretase [45], while another showed that the Aβ-inhibiting effect of DHA is mostly mediated by affecting presenilin 1 rather than the whole γ-secretase complex [26]. A recent study reported that DHA inhibits Aβ accumulation through its hydrophobic characteristics, which leads to the formation of unstructured oligomers [46]. Another possible mechanism involved in the DHA-mediated decrease in Aβ accumulation could be related to the hydrotropic property of ATP [13]. Reportedly, the preventive effect of ATP on Aβ aggregation correlates with ATP concentration [47]. Since DHA treatment significantly improved ATP-linked respiration following mitochondrial dysfunction induced by Aβ (Figure 3C), it is speculated that enhancement of mitochondrial function by DHA may contribute to the improvement of Aβ pathology.

In the progression of AD pathology, neuroinflammation triggered by both microglia and astrocytes is considered one of the cardinal features. In particular, it is thought that Aβ mediates the activation of glial cells [3,4]. Although microglia and astrocytes play considerable roles in the clearance of Aβ, it is also known that glia-induced neuroinflammation leads to neurodegeneration in AD [48]. Moreover, activated glial cells cause neuronal loss via neuroinflammatory responses [49]. Thus, addressing neuroinflammation should be considered as a critical target for AD treatment. Here, immunofluorescent staining of microglia and astrocytes demonstrated a reduction in neuroinflammation following treatment with DHA in the dorsal subiculum of 5XFAD mice (Figure 6A,B). The anti-neuroinflammatory effect of DHA could be due to multiple factors, including mediation of diminished pro-inflammatory cytokines as well as elevated anti-inflammatory cytokines, or an anti-oxidant effect [50]. Interestingly, a recent report on the association between neuroinflammation and mitochondrial dysfunction suggests that mitochondrial-derived damage-associated molecular patterns (DAMPs) stimulate glial cells [51]. In addition, the release of mitochondrial-derived DAMPs, such as cytochrome c and mitochondrial transcription factors, may mediate cellular damage with glial cell activation in the neuroinflammation of neurodegenerative diseases, including AD [52]. Thus, the protective effects of DHA on neuroinflammation in AD might be mediated by multiple pathways, including upstream events during neuroinflammation.

In the present study, DHA attenuated synaptic loss in 5XFAD mice and neuronal loss in both 5XFAD and fat-1 mice (Figure 2 and Figure 6C,D). Evidence from numerous studies has indicated that neuronal cell death and synaptic loss are caused by multiple factors, including Aβ itself and Aβ-mediated neuroinflammation [53,54]. In addition, synaptic dysfunction substantially contributes to cognitive impairment in AD [55,56]. Previous findings may corroborate the effect of DHA on neuronal and synaptic loss [57]. Dietary supplementation of omega-3 PUFAs, containing DHA, improved SYN staining intensity as well as neuronal size in the hippocampal CA1 region in rats by increasing the content of omega-3 PUFAs in the brain [57]. Considering that dietary supplementation of DHA increases the proportion of DHA in the hippocampus of senescence-accelerated prone 8 (SAMP8) mice [58], increased DHA in the central nervous system (CNS) might directly exert a protective effect on neuronal and synaptic dysfunction in AD. Notably, neuronal and synaptic lesions are associated with mitochondrial dysfunction as well as Aβ neurotoxicity [59,60,61]. A recent study demonstrated that DHA inhibits oxidative stress and apoptosis, possibly by ameliorating mitochondrial dysfunction in a brain injury model [62]. Thus, it can be hypothesized that the aforementioned anti-oxidative and anti-apoptotic mechanisms might therefore contribute to preventing neuronal and synaptic loss via modulation of mitochondrial dysfunction. A more direct mechanistic study reported that DHA reduces oxidative stress induced by H_2_O_2_ exposure via upregulation of nuclear factor (erythroid-derived 2)-like 2, a transcription factor regulating antioxidant responses, and heme-oxygenase-1 [52], which supports our study. Although the mechanisms underlying the protective effect of DHA on Aβ-associated neurotoxicity are relatively well established [63], there are limited studies defining the mechanism of action of DHA on mitochondrial dysfunction. Therefore, further studies should be conducted to identify the mechanisms underlying DHA-mediated neuroprotective effects. In the present study, adult hippocampal neurogenesis impairment was recovered by DHA administration in 5XFAD mice (Figure 6E,F). Adult hippocampal neurogenesis, characterized by the formation of new neurons in the DG of the hippocampus, is known to play a critical role in cognitive function. In a recent study, reduced adult hippocampal neurogenesis was observed in the AD brain [64]. A study examining the beneficial effects of omega-3 PUFAs on adult hippocampal neurogenesis indicated that the increased adult hippocampal neurogenesis might be induced by the modulation of neuronal membrane structures, cytokines, and neurotrophins [65]. In addition, more recent evidence demonstrates that adult hippocampal neurogenesis and neuritogenesis are upregulated in fat-1 mice exhibiting increased DHA content [30]. Thus, it can be speculated that the aforementioned mechanisms might be involved in DHA-mediated recovery of adult hippocampal neurogenesis impairment in AD. Another interpretation of the beneficial effect of DHA on adult hippocampal neurogenesis might be related to the effect of omega-3 PUFAs on mitochondrial functions. DHA attenuates oxidative stress in subarachnoid hemorrhage via modulation of mitochondrial dynamics [62]. Furthermore, triglyceride emulsions of DHA ameliorate oxidative stress and improve the mitochondrial Ca^2+^ buffering capacity in hypoxia/ischemia-induced brain injury [66]. This suggests that DHA protects against reduced adult hippocampal neurogenesis in AD via improvement of mitochondrial dysfunction. Additionally, further studies using other markers such as Nissl may be warranted to confirm the neuronal loss.

Considering the evidence highlighting the importance of mitochondrial function in AD pathogenesis [13], our findings demonstrating the protective effect of DHA on mitochondrial dysfunction are noteworthy and have direct implications for the establishment of a treatment for AD. Moreover, we provide evidence that DHA ameliorates Aβ accumulation and Aβ-related pathogenesis. Taken together, the findings of the present study provide important evidence for the development of therapeutic approaches that incorporate the maintenance of mitochondrial function in the AD brain. However, other factors affecting AD pathology—such as Aβ fibrillation, expression levels of presenilin 1, and microglial phagocytosis—also need to be examined. Moreover, given that animal models of AD, such as the 5XFAD mouse used in present study, show some differences compared to human AD pathology [67], deliberate consideration should be given when translating the present results to clinical research. In addition, further studies should be conducted to identify the specific molecular mechanisms that are involved in the DHA-mediated protective effects in the AD brain.

## 4. Materials and Methods

### 4.1. Animals

The fat-1 transgenic mouse can endogenously produce omega-3, due to expression of the fat-1 gene that converts omega-6 into omega-3 [28]. The C57BL/6 mice used here as the control (genetic background) group of fat-1 mice were purchased from Jackson Laboratory (Bar Harbor, ME, USA). Four-month-old male fat-1 (*n* = 8) and C57BL/6 (*n* = 10) mice were used. The 5XFAD mice were purchased from Jackson Laboratory (Bar Harbor, ME, USA) and were cross-bred with male 5XFAD mice and female B6SJL/F1 mice to obtain littermates. The 5XFAD transgenic mice possess both APP mutations (V717I, I716V, K670N/M671L) and PSEN1 mutations (M146L, L286V), which result in characteristic Aβ overexpression. Mice had freely available food and water and were bred in a space with a 12/12 h dark/light cycle. The number of animals was calculated in accordance with resource equation [68,69,70]. Animals were raised according to the National Institutes of Health (NIH) guidelines for the care and use of laboratory animals (NIH Publications No. 8023, revised 1978), and all experiments were approved by the Institutional Animal Care and Use Committee at Konyang University (P-18-03-A-01, approved on 01 August 2018).

### 4.2. Polymerization Chain Reaction for Genotyping

Genomic DNA was extracted from tail samples using the HotSHOT (hot sodium hydroxide and tris) method [71]. For the genotyping of fat-1 and 5XFAD mice, PCRs were performed using mouse genomic DNA and gene-specific oligonucleotide primers (fat-1 forward: 5′-CTG CAC CAC GCC TTC ACC AAC C-3′, fat-1 reverse: 5′-ACA CAG CAG CAG ATT CCA GAG ATT-3′, APP forward: 5’-AGG ACT GAC CAC TCG ACC AG-3’, APP reverse: 5’-CGG GGG TCT AGT TCT GCA T-3’, PSEN1 forward: 5’-AAT AGA GAA CGG CAG GAG CA-3’, PSEN1 reverse: 5’-GCC ATG AGG GCA CTA ATC AT-3’) in the AccuPower^®^ HotStart PCR premix (Bioneer, Daejeon, Republic of Korea) according to the manufacturer’s protocol (Figure S4). The polymerization reactions were incubated at 94 °C for 3 min, followed by 35 cycles of 94 °C for 30 s, 54 °C for 20 s, and 72 °C for 30 s, then followed by 72 °C for 2 min, in the Labcycler Gradient (SensoQuest GmbH, Göttingen, Germany). The PCR products were loaded onto a 1% agarose gel in the Tris-acetate-EDTA buffer (0.02M Tris-acetate and 0.5 mM EDTA), electrophoresis for 30 min and then visualized with RedSafe™ (iNtRON Biotechnology, Seongnam-si, Korea).

### 4.3. Administration of DHA

To evaluate the efficacy of DHA, 200 mg/kg DHA (cis-4,7,10,13,16,19-docosahexaenoic acid; Sigma-Aldrich, St. Louis, MO, USA) was orally administered every other day for 2 weeks in 3-month-old male 5XFAD and WT mice. The injection regimen was determined based on previous study and by slightly modifying the administration period [72,73]. The mice were divided into three experimental groups: (1) WT+saline (*n* = 7), (2) 5XFAD+saline (*n* = 7), and (3) 5XFAD+DHA (*n* = 7).

### 4.4. Characterization of Aβ Aggregation

Prior to establishing an acute AD model through stereotactic injection of Aβ, aggregation of Aβ was evaluated by a Thioflavin T (ThT) assay (Figure S5). Aβ_1–42_ peptide (Bachem, Bubendorf, Switzerland) was solubilized in 0.1 M aqueous ammonia solution to prepare a 27.5-μM Aβ peptide solution. ThT (Tokyo Chemical Industry, Tokyo, Japan) was dissolved in 50 mM glycine buffer to prepare a 15-μM ThT solution. A final 100 μL reaction solution was made, consisting of 67.5 μL 27.5-μM Aβ peptide solution, 25 μL 15-μM ThT solution, and 7.5 μL of distilled water. As a positive control to inhibit Aβ oligomerization, 2 mM Morin (Sigma-Aldrich, St. Louis, MO, USA), an inhibitor of Aβ oligomerization [74], was used instead of triple distilled water. The ThT reaction solution was incubated in a 96-well black plate (SPL life Sciences, Pocheon-si, Republic of Korea) for 1, 2, 3, and 4 h at 37 °C, after which the fluorescence intensity of ThT was measured at Ex/Em = 440 nm/484 nm using a SpectraMax iD3 Multi-Mode Microplate Reader (Molecular Devices, San Jose, CA, USA).

### 4.5. Stereotaxic Surgery

It has been reported that 5XFAD mice show rapid and massive Aβ_1–42_ accumulation [32]. In addition, monomeric Aβ_1–42_ results in Aβ accumulation by increasing expression of BACE1 and its activity [75]. To examine the effect of endogenous elevation of omega-3 PUFAs on Aβ-induced toxicity, stereotaxic surgery was conducted in anesthetized 4-month-old male fat-1 and C57BL/6 mice. Stereotaxic injection of Aβ was performed as previously described [76,77], using 10 μM (3 μL) of Aβ oligomer. Considering that in vivo clearance of monomeric Aβ is higher than that of oligomeric Aβ [78], we adjusted the concentration of the monomeric Aβ for stereotactic injection to 100 μM (1.5 μL). 100 μM of Aβ_1–42_ or saline (vehicle) were injected for 3 min at a rate of 0.5 μL/min into the hilus of the hippocampal DG in the both fat-1 and C57BL/6 mice using a Hamilton syringe. The bilateral coordinates used for the stereotaxic surgery were AP −2.0 mm, ML ± 1.5 mm, and DV −2.0 mm based on bregma. The coordinates for stereotactic injection into the hilus of DG were established referenced on the Paxinos and Franklin’s *The Mouse Brain in Stereotaxic Coordinates* [79], and the coordinates were verified by injection of 0.1% cresyl violet solution (Sigma-Aldrich, St. Louis, MO, USA) (Figure S6). After the injection, the syringe remained in place for 3 min to prevent backflow of the solution while removing the needle.

### 4.6. Preparation of Brain Tissue

The fat-1 and C57BL/6 mice were sacrificed 7 days after stereotaxic surgery. The 5XFAD and WT mice were sacrificed 1 day after the final administration of DHA. Mice were anesthetized and then cardiac-perfused with 0.05 M phosphate-buffered saline (PBS) containing 4% paraformaldehyde (PFA). The brains of cardiac-perfused mice were isolated and post-fixed in 4% PFA for 20 h at 4 °C, and then immersed in 0.05 M PBS containing 30% sucrose for 3 days to prevent damage upon freezing. The brains were sectioned in the coronal plane using a cryostat (Leica Biosystems, Wetzlar, Germany) to a thickness of 30 μm and incubated in storing solution (25% glycerol, 25% ethylene glycol, in 0.05 M PB) at 4 °C until the tissues were used.

### 4.7. Cell Culture and DHA Treatment

The mouse hippocampal neuronal cell line (HT22) was cultured in Dulbecco’s modified Eagle’s medium (DMEM; WELGENE, Gyeongsan-si, Republic of Korea) containing 10% fetal bovine serum (FBS; Gen DEPOT, Katy, TX, USA) and 0.1 mg/mL of penicillin-streptomycin at 37 °C with 5% CO_2_. The HT22 cells were counted (3.2 × 10^3^ cells/well) and seeded onto a Seahorse XFp cell culture plate (Seahorse Bioscience, Billerica, MA, USA). Twenty-four hours later, the cells were treated with DHA (10 μM) and then with Aβ (2 μM; Bachem, Bubendorf, Switzerland) after 1 h, followed by XFp analysis.

### 4.8. Cell Viability Assay

An MTT (3-(4,5-dimethylthiazol-2-yl)-2,5-diphenyltetrazolium bromide; Sigma-Aldrich, St. Louis, MO, USA) assay with mitochondrial reductase was used to measure cell viability. HT22 cells were transferred to a 96-well microplate at 5 × 10^3^ cells per well in DMEM medium containing 10% FBS and incubated for 24 h. HT22 cells were then treated with DHA (1, 10, 20, 30, 40, and 50 μM) for 24 or 48 h. MTT solution was added at a concentration of 0.5 mg/mL per well and left to cultivate for 2 h at 37 °C. After discarding the medium containing the MTT solution, attached HT22 cells were incubated with dimethyl sulfoxide to elute the reduced formazan crystals for 1 h at 37 °C; then absorbance was measured at 540 nm.

### 4.9. Measurements of Oxygen Consumption Rate

The OCR was detected using a Seahorse XFp Cell Mito Stress Test Kit (Seahorse Bioscience, Billerica, MA, USA) with glucose (4.5 mg/mL), L-glutamine (0.584 mg/mL), and pyruvate (0.11 mg/mL) added to the Seahorse XF base medium (Seahorse Bioscience, Billerica, MA, USA). The OCR measurements using the Seahorse XFp Cell Mito Stress Test Kit were performed following the manufacturer’s protocol. First, the kit had to be calibrated using the loaded assay cartridge. The machine recorded the O_2_ concentration every 4 min in pmol/min. After the measurement of baseline rates, preloaded oligomycin (1 μM; Seahorse Bioscience, Billerica, MA, USA), FCCP (1 μM; Seahorse Bioscience, Billerica, MA, USA), rotenone (0.5 μM: Seahorse Bioscience, Billerica, MA, USA), and antimycin A (0.5 μM; Seahorse Bioscience, Billerica, MA, USA) were released consecutively into each well in a calibration chamber. The OCR was recorded three times after mixing the injected drugs and assay medium for 6 min. The averages of three baseline rates and three test rates were analyzed along with the machine’s automatically generated one-page assay summary.

### 4.10. Immunohistochemistry

Three sections per brain were obtained from the hippocampus (between −1.3 mm to −2.3 mm from bregma) and the subiculum (between −2.6 mm to −4.3 mm from bregma) for immunohistochemical staining with each antibody. Primary antibodies against mouse anti-Tom20 (WH0009804M1, 1:200, Sigma-Aldrich, St. Louis, MO, USA), mouse anti-SYN (S5768, 1:500, Sigma-Aldrich, St. Louis, MO, USA), mouse anti-NeuN (MAB377, 1:1,000, Merck Millipore, Burlington, MA, USA), mouse 4G8 (SIG-39220, 1:2,000, Biolegend, San Diego, CA, USA), goat anti-Iba-1 (ab5076, 1:500, Abcam, Cambridge, UK), rat anti-GFAP (13-0300, 1:200, Thermo Fisher, Waltham, MA, USA), rabbit anti-Ki-67 (ab15580, 1:500, Abcam, Cambridge, UK), and goat anti-doublecortin (DCX) (sc-271390, 1:200, Santa Cruz Biotechnology, Dallas, TX, USA) were prepared in PBS containing 0.5 mg/mL BSA and 0.3% Triton X-100 at the respective dilution ratios. The brain tissue was incubated in primary antibody overnight at 4°C, and the submerged tissue was washed three times for 5 min in PBS. Next, the brain tissues were incubated in secondary antibody for 1 h at room temperature. Secondary antibodies (1:200, Thermo Fisher, Waltham, MA, USA) including: donkey Alexa Fluor^®^ 594-conjugated anti-goat antibody, donkey Alexa Fluor^®^ 594-conjugated anti-rabbit antibody, donkey Alexa Fluor^®^ 594-conjugated anti-rat antibody, goat Alexa Fluor^®^ 488-conjugated anti-mouse antibody, donkey Alexa Fluor^®^ 488-conjugated anti-mouse antibody, and donkey Alexa Fluor^®^ 488-conjugated anti-goat antibody were constructed in PBS with 0.3% Triton X-100. Validation of immunohistochemistry was performed in the absence of primary antibody (Figure S7). The brain tissues were incubated in the secondary antibody and washed three times in PBS, mounted on SuperFrost Plus™ slides (Thermo Fisher, Waltham, MA, USA), and then covered with Fluoroshield™ with 4′,6-diamidino-2-phenylindole (DAPI; Sigma-Aldrich, St. Louis, MO, USA).

### 4.11. Image Acquisition and Analysis

Images were acquired from the immunostained brain tissues using a Zeiss LSM 700 microscope (Carl Zeiss AG, Oberkochen, Germany), and the images were analyzed using ImageJ software (NIH, Bethesda, MD, USA). Histological analysis of Tom20 was performed as previously described [8,31]. The percentage of the Tom20-positive area and the fluorescence intensity were measured using ImageJ software. To assess mitochondrial dynamics, the total fragmentation of the Tom20-positive area was measured after setting this area as the threshold. In addition, the filamentous structure (≥2 μm to infinity) and small pieces (≥0.1 μm to <2 μm) were calculated for each area and sorted according to the Tom20-positive area size. The total number of fragments per area and the average size of the total fragments were quantified using ImageJ software. Finally, the percentage of small fragmentation and filamentous structures in the total number of fragments was calculated. For the quantification of GFAP, Iba-1, and 4G8 immunoreactivity, the area fraction for immuno-positive signals was quantified in each brain section. The GFAP, Iba-1, and NeuN immunoreactivity were quantified as the total number of immuno-positive cells per area of brain region (mm^2^). The immunoreactivity of SYN was quantified as the fluorescence intensity in the dorsal subiculum. The number of DCX or Ki67 (+) cells was quantified as the total number of immuno-positive cells per length of the subgranular zone (SGZ) in the DG. Image obtainment, histological quantification, and statistical analysis were performed in a blind manner for each group.

### 4.12. Statistical Analysis

All statistical analyses were conducted using GraphPad Prism 7.0 software (GraphPad Software, Inc., La Jolla, CA, USA). Data are presented as mean ± standard error of the mean (SEM). The Kolmogorov–Smirnov test was performed for normality validation. When the Kolmogorov–Smirnov test showed no significant differences, the Levene test was conducted to analyze variances between groups. An unpaired *t*-test with Welch’s correction was used for comparison between the two groups (5XFAD + vehicle and 5XFAD + DHA). For statistical analysis between three or more groups, one-way ANOVA with Tukey’s post hoc test was used. For the analysis of variables not fulfilling the normality distribution, Kruskal–Wallis test with Dunn’s post hoc test was used. A *p*-value of < 0.05 indicated statistical significance.

## 5. Conclusions

**Figure 7:**
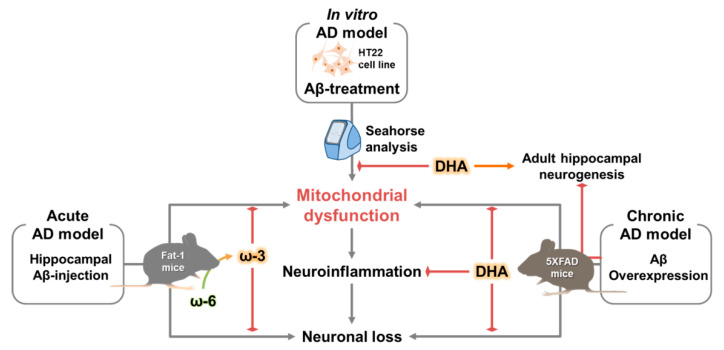
Schematic illustration of the effect of omega-3 fatty acid-type docosahexaenoic acid on AD-related pathology. Arrow-headed lines: stimulation, bar-headed lines: inhibition.

The present study suggests that the omega-3 fatty acid DHA reduces Aβ accumulation, neuroinflammation, and neuronal loss and ameliorates adult hippocampal neurogenesis impairment by protecting against mitochondrial dysfunction in AD pathogenesis (Figure 7). These beneficial effects of DHA could be applicable in the establishment of mitochondria-targeted approaches for AD treatment.

## Supplementary Materials

Supplementary materials can be found at https://www.mdpi.com/1422-0067/21/11/3879/s1.

## Abbreviations

AD	Alzheimer’s disease
Aβ	Amyloid-beta
DCX	Doublecortin
DG	Dentate gyrus
DHA	Docosahexaenoic acid
DPA	Docosapentaenoic acid
EPA	Eicosapentaenoic acid
GFAP	Glial fibrillary acidic protein for astrocyte
Iba-1	Ionized calcium-binding adapter molecule 1
NeuN	Neuronal nuclei
PUFAs	Polyunsaturated fatty acid
SGZ	Subgranular zone
SYN	Synaptophysin
Tom20	Translocases of mitochondrial outer membrane 20
