# Supplementary material for: Omega-3 Fatty Acid-Type Docosahexaenoic Acid Protects against Aβ-Mediated Mitochondrial Deficits and Pathomechanisms in Alzheimer’s Disease-Related Animal Model
Source: Int J Mol Sci. 2020 May 29;21(11):3879. doi: 10.3390/ijms21113879 (PMC7312360; doi:10.3390/ijms21113879)
Supplement: Supplementary file 1 [file ijms-21-03879-s001.pdf]

Supplementary figure

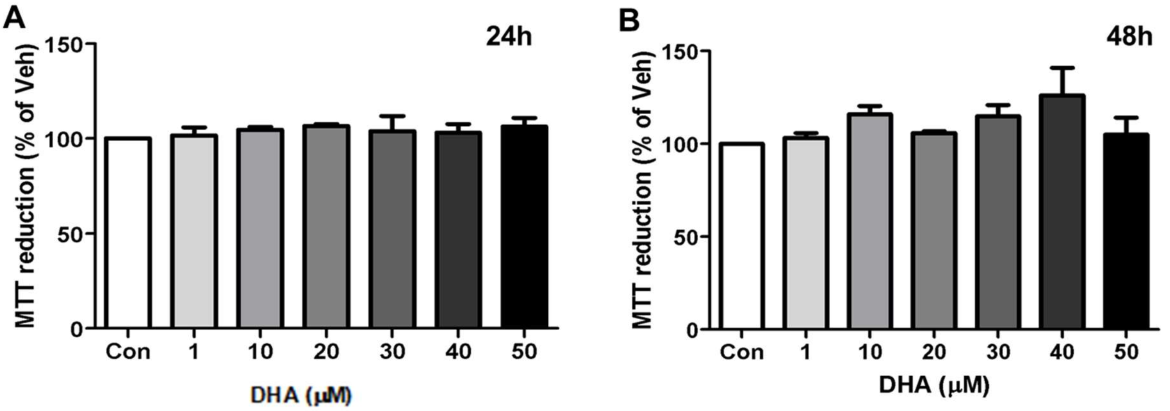

Figure S1. Cytotoxicity evaluation of DHA in HT22 cell line through MTT assay. HT22 cells were treated with 1, 10, 20, 40, or 50 μM DHA and MTT reduction was analyzed after 24 (A) or 48 (B) h.

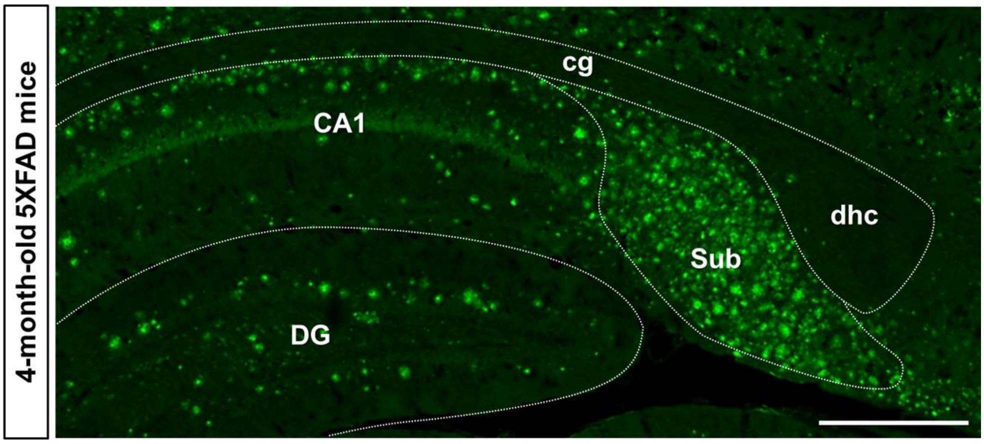

Figure S2. Distribution of Aβ plaque in the hippocampal formation of 5XFAD mice. Aβ plaques were visualized in the hippocampus of 5XFAD mice using 4G8 antibody. Sub: Subiculum, CA1: hippocampal field CA1 of hippocampus, DG: dentate gyrus, cg: cingulum, dhc: dorsal hippocampal commissure.

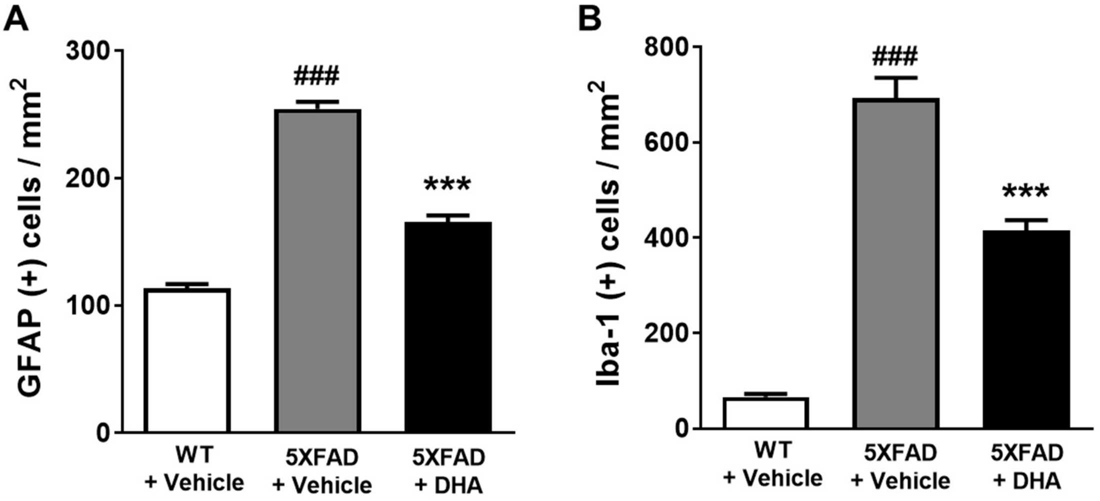

Figure S3. Quantification of Iba-1 and GFAP in the subiculum of DHA-administered 5XFAD mice. As a result of quantifying the numbers of Iba-1 (A) and GFAP (B) positive cells per area of the dorsal subiculum, both significantly upregulated numbers of Iba-1 and GFAP (+) cells in the 5XFAD mice were significantly alleviated by administration of DHA. Each histological qualitative data are presented as mean ± SEM (42 images / 7 mice). $^{###}p < 0.001$: vehicle-treated WT mice versus vehicle-treated 5XFAD mice. $^{***}p < 0.001$: vehicle-treated 5XFAD mice versus DHA-treated 5XFAD mice.

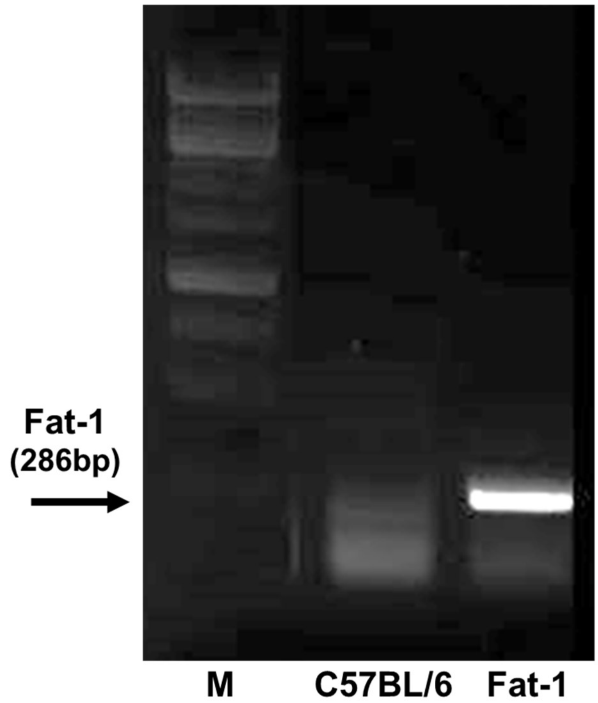

Figure S4. Validation of fat-1 transgene expression in C57BL/6 and fat-1 mice by PCR.

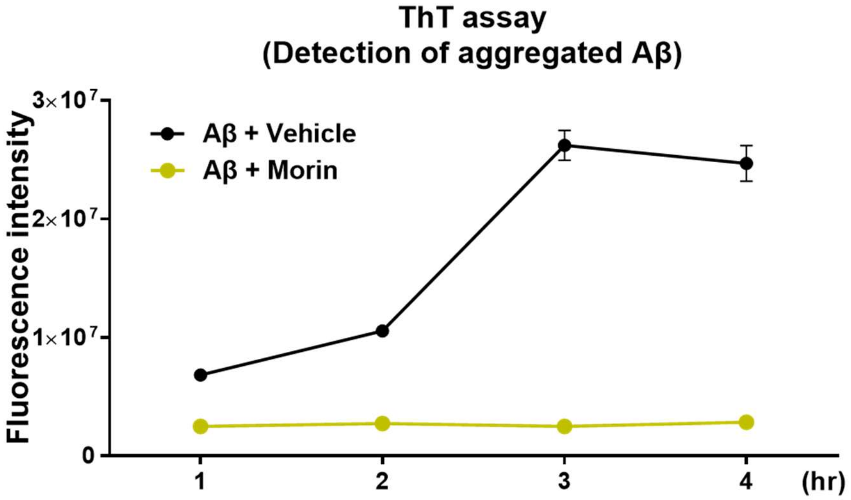

Figure S5. Characterization of Aβ aggregation. ThT assay showed the aggregation of Aβ over time from 1-4 hours as the intensity of fluorescence. Morin was used as an indicator for anti-aggregation of Aβ. Inhibitor of Aβ oligomerization, morin inhibited the self-aggregation of monomeric Aβ, resulting to maintaining the unaggregated Aβ.

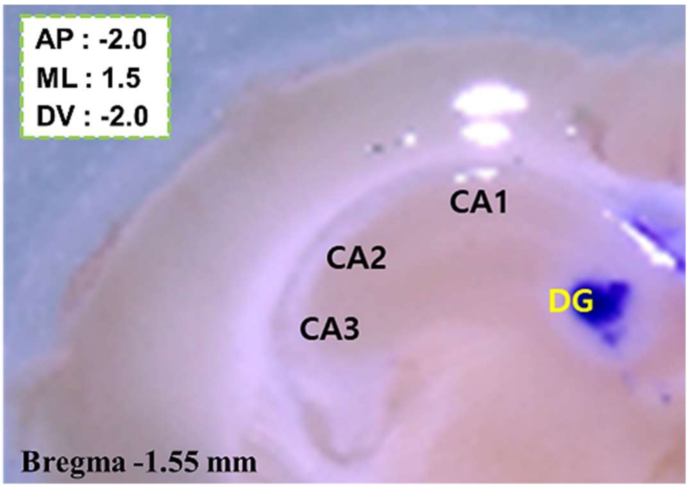

Figure S6. Validation of the stereotaxic injection in to the hilus of dentate gyrus. 1.5 μl of 0.1% cresyl violet (CV) solution was injected according to stereotactic coordinates (AP -2.0 mm, ML + 1.5 mm, and DV -2.0 mm) for 3 min at a rate of 0.5 μl/min. After injection, the mouse was sacrificed to extract the brain. the injected CV observed at a coronal section from bregma -1.55 mm. CA1-3: hippocampal field CA1-3 of hippocampus, DG: dentate gyrus.

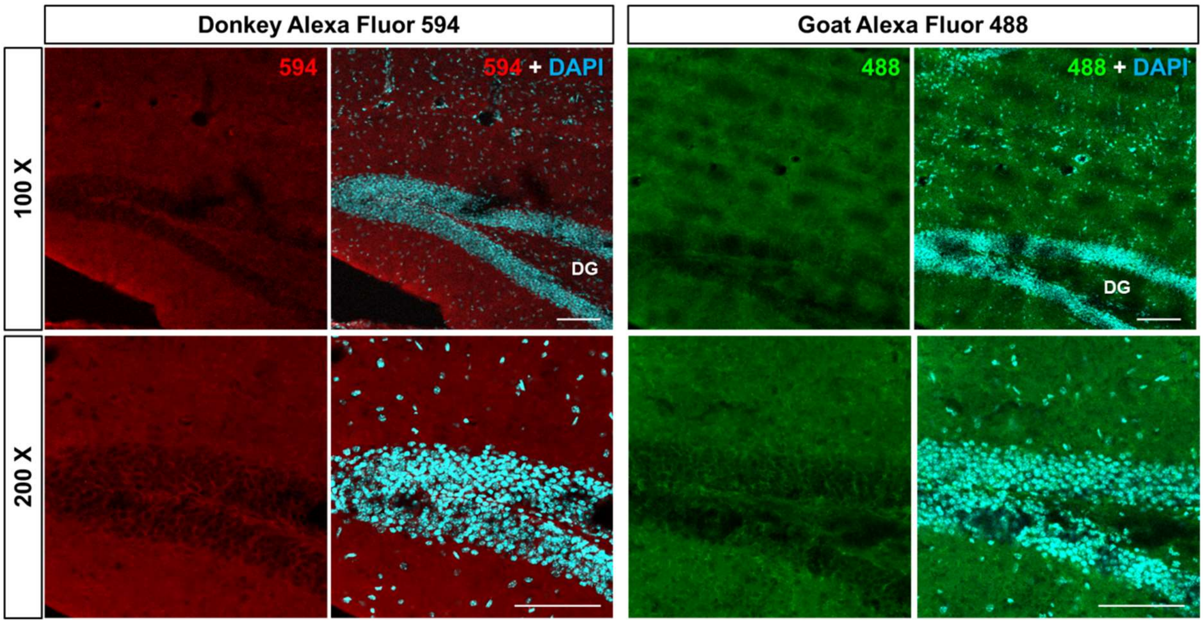

Figure S7. Validation of the immunohistochemistry. According to the immunohistochemical procedure, brain tissues were cultured in the absence of primary antibodies, followed by culturing fluorescence-conjugated secondary antibodies 1) Donkey Alexa Fluor 594 (left) or 2) Goat Alexa Fluor 488 (right) to obtain primary antibody negative control staining. Verify that there is no non-specific signal in the absence of primary antibody.
